# Implementation of a pharmacist‑led discharge intervention to improve medication information handover at hospital discharge: a non-randomised feasibility study

**DOI:** 10.1186/s12913-026-14940-1

**Published:** 2026-06-17

**Authors:** Matt Percival, Brigid M. Gillespie, Hayley Hirsch, Kate Johnston, Georgia Tobiano, Salim Memon, Noela Baglot, Rohan Jayasinghe, Carl de Wet, Mark Morgan, Laetitia Hattingh

**Affiliations:** 1 School of Pharmacy and Medical Sciences, Griffith University, Southport, QLD Australia; 2 School of Pharmacy, The University of Queensland, Brisbane, QLD Australia; 3 Integrated Care Services, Gold Coast Hospital and Health Service (GCHHS), Southport, QLD Australia; 4 School of Nursing and Midwifery, Griffith University, Southport, QLD Australia; 5 Nursing and Midwifery Education and Research Unit, GCHHS, Southport, QLD Australia; 6 Pharmacy Department, GCHHS, Southport, QLD Australia; 7 GP Partnerships and Engagement, GCHHS, Southport, QLD Australia; 8 NHMRC CRE in Wiser Wound Care, Griffith Health, Griffith University, Southport, QLD Australia; 9 Strategy, Transformation and Major Capital, GCHHS, Southport, QLD Australia; 10 Consumer Advisory Group, GCHHS, Southport, QLD Australia; 11 Cardiology Department, GCHHS, Southport, QLD Australia; 12 Medical Services and Clinical Governance, South-West Hospital and Health Service, Roma, QLD Australia; 13 Faculty of Health Sciences and Medicine, Bond University, Robina, QLD Australia; 14 Allied Health Research, Gold Coast Hospital and Health Service (GCHHS), Southport, QLD Australia; 15 Robina Health Precinct, 2 Campus Crescent, Robina, QLD 4226 Australia

**Keywords:** Medicine-related harm, Transitions of care, Pharmacist-led medication reconciliation, Collaborative pharmacist medication prescribing, RE-AIM framework

## Abstract

**Background:**

Transitions of care have been identified as high-risk periods for medication-related harm. This feasibility study assessed the reach, adoption, implementation, maintenance and acceptability of a multifaceted intervention designed to improve medicine information handover (patient medicine lists and discharge medicine reconciliation) at hospital discharge in a large public health service in southeast Queensland, Australia.

**Methods:**

A non-randomised mixed methods study was conducted, guided by RE-AIM framework. Our primary aim was to determine whether the intervention and study procedures were practical, acceptable, and deliverable in the real-world setting. The population were patients ≥ 65 years of age admitted to hospital for > 24 h on one of three medical wards. The intervention included digital risk stratification, documentation of reasons for medication changes within an electronic medical record, web and paper-based patient information resources, and pharmacist engagement with discharge medications. Quantitative data was used to present recruitment and consent rates, and patient responses to post-discharge survey questions, whilst brief and semi-structured interviews explored clinician participants’ perspectives. Inductive content analysis was used on all interview transcripts with deductive mapping to the RE-AIM framework.

**Results:**

Of 36 patients approached, 17 (47%) provided consent and 12 (33%) completed surveys. All invited doctors (*n* = 20) and pharmacists (*n* = 3) participated in the intervention. Of the 12 patients surveyed, 11 (92%) felt included in medication-related decisions; nine (75%) followed up with their general practitioner post-discharge, and five (56%) did so within one week. Nine doctors and three pharmacists were interviewed. Clinicians viewed the intervention positively, citing improved workflow, reduced workload and enhanced learning opportunities. Barriers included variable uptake across medical teams, and a need for improved communication and training. Participant feedback suggested the intervention would be sustainable long term once overcoming initial challenges.

**Conclusion:**

The pharmacist-led, multifaceted intervention was feasible and well received by patients, doctors and pharmacists. It improved communication, workflow and shared learning, supporting pharmacist-led medication reconciliation as a scalable model for improving medicine handover. Larger, multi-site evaluations with extended follow-up are warranted to confirm generalisability and effectiveness.

**Trial registration:**

This was a feasibility study that did not meet the definition of a clinical trial under the International Committee of Medical Journal Editors (ICMJE) and was not registered as such.

**Supplementary Information:**

The online version contains supplementary material available at https://doi.org/10.1186/s12913-026-14940-1.

## Introduction

### Background/rationale

Medication-related harm is defined by the World Health Organization (WHO) as the “harm caused by medication if taken incorrectly, monitored insufficiently or as the result of an error, accident or communication problem.” [1] Globally, costs associated with medication errors have been estimated at $42 billion USD annually [1]. In Australia, it is estimated that 250,000 hospital admissions occur annually due to medication related harm, with an estimated cost of 1.4 billion Australian dollars [2]. The impact of medication-related harm on patients is considerable and can range from mild harm to severe life threatening events [3, 4], psychological tolls such as loss of confidence, anxiety and fear of recurrence [5], and reduction in functional capacity through increased risk of falls, fractures, reduced mobility and cognitive impairment [6]. Older patients are particularly at risk of medication related harm due to factors such as polypharmacy associated with multimorbidity, age-related changes in pharmacokinetics and dynamics, and challenges with medication adherence [7, 8].

Transitions of care (TOC) have been identified as high-risk periods for medication related harm for older people [9]. Examples of medication related harm during TOC can include incorrect medication directions provided to patients (e.g., methotrexate daily instead of weekly) or an omission of an important medication as the patient is discharged back to primary care (e.g., insulin in a person with diabetes). Patients discharged from hospital back to the community setting represent one such example of TOC, with an estimated 51% of older adults experiencing medication related harm post hospital discharge with 35–59% of events considered preventable [10]. Older patients may be confused about medicine changes whilst in hospital and unintentionally double-up on doses and hence overdose if not realising new medicines are the same as what they already have at home or miss doses when they do not have sufficient medicines post-discharge. Therefore, healthcare facilities have focused on interventions to reduce the risk of medication related harm during care transitions [1].

Key interventions to reduce medication related harm identified in the literature have included patient education and counselling on discharge, identification and resolution of medication discrepancies, medication reconciliation, pharmacist-led interventions, use of information technology, discharge planning, improved communication across TOC, and patient follow up [11–18]. Delivering a combination of these interventions, i.e. through a pharmacist-led, multi-faceted intervention across the discharge period, has been more successful compared to isolated interventions [14, 19].

Recognising ongoing challenges with medication-related harm at discharge, the Gold Coast Hospital and Health Service (GCHHS) developed a pharmacist-led intervention to improve medication handover for older adults [20–22]. The GCHHS encompasses two hospital campuses and four community health centres, situated in southeast Queensland, Australia. The intervention was conceived in two phases, published previously [20, 21], with a third phase implementing the intervention in a pilot study, also published previously [22]. Briefly, Phase 1 used contextual inquiry through nominal group technique workshops to rank discharge medication handover issues and solutions, involving hospital clinicians, hospital leadership, primary care providers and consumers. Phase 2 incorporated workshops, interviews and stakeholder consultation using a co-design process to refine and develop a multifaceted intervention to improve medication handover from hospital at discharge. This led to the development of a pharmacist-led, multifaceted intervention composed of four components that included (i) tools to support patient engagement in medications at discharge, (ii) validated patient risk stratification tool to assist health care professionals prioritise patients at risk of medication related harm, (iii) training doctors to document reasons for medication changes in the integrated electronic medical record (iEMR), and (iv), collaborative pharmacist medication prescribing (CPMP) during discharge preparation. Phase 3 consisted of a pilot study to evaluate and assess the preliminary effects of the pharmacist-led, multifaceted intervention on discharge medicine information handover from hospital to primary care [22]. Inclusion criteria for the Phase 3 component were patients ≥ 65 years admitted to hospital for > 24 h with a pharmacist documented admission history note completed before discharge. Results from this Phase 3 component, demonstrated the pharmacist-led, intervention increased the proportion of electronic discharge summaries containing a list of patient medicines, halved the time taken for general practitioners to receive patient discharge summaries, and showed > 30 min reduction in the time discharges were confirmed and patients able to leave hospital [22]. These results align with the Australian Commission on Safety and Quality in Health Care recommendations of timely, accurate discharge summaries to prevent medication related harm at transitions of care [23].

This initiative addresses a critical gap in transitional care by integrating patient engagement, documentation of prescribing rationale, risk stratification to prioritise patients at higher risk of medication related harm, and pharmacist-led medication reconciliation. While developed within GCHHS, the intervention has broader relevance for health services nationally and internationally, offering a scalable model to enhance medication safety and continuity of care during hospital discharge—a period consistently associated with elevated risk of medication related harm.

The importance of the current study is to assess the feasibility of the pharmacist-led, multifaceted intervention, to ensure that the benefits of this innovative model of care can be translated into real-world contexts. Too often, interventions fail to progress into practical settings due to absence of proper feasibility testing, Our feasibility study will help inform a future, larger study and assist with identifying ways to successfully implement the intervention in a real world setting.

In this paper, we assessed the feasibility of the above mentioned pharmacist-led intervention, for future broader implementation and scalability using the RE-AIM framework [24] with a focus on its five dimensions of reach, efficacy, adoption, implementation, maintenance [24] and acceptability of the intervention [25–27]. The RE-AIM framework provides an approach to determine whether a program or intervention can function in a ‘real world’ setting and assesses whether such a program is worthy of further investment from individual and organisational levels. The RE-AIM framework offers an approach to assess the feasibility of complex interventions in real world settings that fall outside of the highly controlled, randomised controlled trial. The RE-AIM framework includes five dimensions: reach, efficacy, adoption, implementation and maintenance [24, 28].

- Reach: patient level engagement, i.e., proportion of eligible patients who participated in the pharmacist-led intervention or accessed the patient engagement, medication management tools.
- Efficacy: impact of the intervention on specified criteria when implemented as intended. It should be noted that this *efficacy* component has already been evaluated and previously reported [22], described above.
- Adoption: provider or organisational level uptake, i.e., proportion of inpatient units, doctors and pharmacists who implemented the intervention.
- Implementation: intervention fidelity and the extent to which the intervention was implemented and adhered to in a real-world setting.
- Maintenance: perceived sustainability and ongoing integration into the work environment, as reported by pharmacists and doctors.

The acceptability of the pharmacist-led intervention to both patients and health care professionals is also an essential element in ensuring longevity and was also included as part of our feasibility assessment.

### Aim

This study aimed to assess the feasibility of the pharmacist‑led, mutlifaceted intervention and study procedures, specifically whether they were practical, acceptable, and deliverable in a real‑world setting. Feasibility of the intervention is described in the context of the RE-AIM framework, with a focus on reach, adoption, implementation, maintenance [24] and acceptability of the intervention [25–27]. The acceptability of the pharmacist-led intervention to both patients and health care professionals was also an essential element in ensuring longevity and was included in our feasibility assessment.

## Method

### Study design

This study design is based on a non-randomised, mixed-methods design, focusing on assessing the feasibility of implementing a pharmacist-led, multifaceted intervention described above. Ethics approval was received from the Gold Coast Hospital and Health Service Human Research Ethics Committee (Ref No. HREC/2023/QGC/94932). The study was conducted in accordance with the National Statement on Ethical Conduct in Human Research (National Health and Medical Research Council, Australia) and the principles of the Declaration of Helsinki. The Consolidated criteria for Reporting Qualitative research (COREQ) guideline was used in conjunction with the Better reporting of interventions: template for intervention description and replication (TIDieR) checklist to ensure clarity in articulating study findings [29, 30]. Informed consent was obtained from all participants, after they were provided with and had the opportunity to review a Participant Information Consent Form (PICF).

### Setting

The pharmacist-led, multifaceted intervention was implemented across three acute GCHHS inpatient units (IPUs): two IPUs at the Gold Coast University Hospital (GCUH), a tertiary referral hospital with approximately 900 beds, and one IPU at Robina Hospital (approximately 400 beds), a major regional hospital within the same health service [21].

### Intervention

This feasibility study was conducted over 9 weeks between 19 June to 28 August 2024 (6-week period at GCUH and 3-week period at Robina Hospital). Patient inclusion criteria were: age ≥ 65 years, hospital admission > 24 h, and prior pharmacist review with a documented medication history in the electronic medical record before discharge. The documented medication history was conducted by a pharmacist at some point during the patient’s hospital admission. The pharmacist-led, multifaceted intervention (previously reported [21, 22]) had four components, presented in Table 1.

**Table 1:** Pharmacist-led, multifaceted intervention

Multifaceted intervention component	Description
1. Patient engagement	Patients were encouraged to ask questions to the pharmacist or other medical staff (nurse or doctors) regarding their medications prior to discharge and were provided with a quick response (QR) code that linked to a (i) question builder website where they could enter questions about their medicines that would be emailed to a pharmacist and (ii) landing page, containing evidence-based medicine resources. Examples of queries patients might ask included, “what are my medications for?”, “what sort of side effects are associated with my medication?”
2. Hospital doctor training to record reasons for medication changes	Doctors were encouraged to document the reasons for medication changes in the integrated electronic medical record to assist ward pharmacists in incorporating this into discharge medication records (or equivalent medicine list).
3. Use of validated risk prediction tool to assist pharmacists stratify patients at risk of medication related harm	Hospital pharmacists identified patients at increased risk of medication-related harm and risk scores determined handover to primary care clinicians at the time of patient discharge from hospital.
4. Pharmacist-led discharge medicine reconciliation planning	Hospital pharmacists collaborated with doctors to generate the discharge medication list. This task was previously performed by doctors prior to implementing the multifaceted intervention.

### Participant recruitment and interview process

Participants (patients, doctors, and pharmacists) were approached in-person by a member of the research team (HH) and invited to participate in interviews to explore their perceptions and experiences of the pharmacist-led, multifaceted intervention. Patients were invited to participate in a brief phone survey one week following their hospital discharge. Closed-ended questions were used but patients could elaborate or expand on their responses. Longer, more in-depth semi-structured interviews were believed to be taxing on patients recovering from a recent hospitalisation, hence a brief phone discussion was considered more appropriate. Given time constraints, brief interviews were conducted face-to-face with doctors, with more detailed, semi-structured interviews with pharmacists. Pharmacist interviews were conducted in greater depth as their role in medication reconciliation and documentation was directly aligned to the study objectives, requiring detailed exploration of workflows and processes. In contrast, doctors were interviewed more briefly due to time constraints and their indirect involvement in the processes under investigation. Doctors and pharmacists consenting to participate in the interviews were interviewed at their regular place of work. All interviews were conducted by one member of the research team (HH).

### Data collection

Quantitative data relating to participant demographics were collected from all participants and extracted from an Excel spreadsheet used by the research pharmacist (HH). Demographic data was collected using the electronic medical record, which provided age, sex, number of medications at discharge, and medication related harm (PRIME score). The PRIME score is a validated risk prediction tool that uses eight variables (age, gender, antiplatelet drug, sodium level, antidiabetic drugs, past adverse drug reaction, number of medicines, living alone) to predict risk of medication related harm in older adults ≥ 65 years following hospital discharge [7]. All variables to calculate the PRIME score were automatically extracted from the electronic medical record into a pharmacist dashboard, except for the “living alone” variable, which needed to be manually entered by the hospital pharmacist to complete the calculation [7]. The questions used in the survey/interviews for each of the participants (patients, doctors and pharmacists) were developed around the facets of the intervention they were involved with. Questions were revised with input from the research team members (LH, BG, HH) and piloted for clarity. Patient phone call responses were recorded by the research pharmacist (HH) whilst on the phone and transcribed immediately after into an excel spreadsheet. Each survey followed a standardized question guide and was capped at approximately 10 min to ensure consistency across patients. However, where patients raised other relevant issues, these were incorporated into subsequent interviews where appropriate. Patients were allowed to elaborate or expand on their responses and were not stopped at the 10 min point if further insights were being shared. Doctors were interviewed face-to-face on the day their participation in the intervention concluded, whilst pharmacists were interviewed either face-to-face or via Microsoft Teams ^®^ one to two weeks following the conclusion of the study. The research pharmacist conducting the interviews (HH) had no prior clinical or supervisory relationship with participants, reducing the risk of socially desirable responses. All interviews were recorded using Microsoft Teams ^®^ or a smart phone, with the audio files transcribed using a professional transcription company. Examples of questions asked of participants are presented below in Table 2, with a full question guide for all participants available in the supplementary material. The research pharmacist kept field notes for their own use throughout the data collection period, collecting data about the reach and adoption of the intervention.

**Table 2:** Examples of questions asked to patients, doctors and pharmacists

Patients	Doctors	Pharmacists
Was the patient information provided helpful whilst in hospital?	Did pharmacist-led discharge medication reconciliation impact your workflow?	In regard to the discharge reconciliation planning, how did this impact your workflow?
Did a GCHHS staff member discuss medication changes with you when you were discharged from hospital?	Did pharmacist-led discharge medication reconciliation impact your workload?	What was the impact on workload?
When medication changes were made in hospital, were you told about them?	Did you feel more confident about discharge recs when you signed them off if the pharmacist had done the plan before?	Did you feel confident taking on this role of discharge rec planning?
Were you given the opportunity to ask questions?	Do you think it helped the timeliness of discharges?	Do you think it helped facilitate the timeliness of discharges?

### Data analysis

Descriptive statistics were used to describe the reach and adoption of the pharmacist-led, multifaceted intervention represented by the number of patients, doctors, and pharmacists participating in the intervention. Patient phone survey responses were also summarised using descriptive statistics, and comments were provided where given.

Free-text comments were documented by the research pharmacist for more context and insight. Data from interviews with doctors and pharmacists were analysed using inductive content analysis, with codes and categories derived directly from the data [31]. To preserve participant perspectives, no predefined framework was used to guide coding. Following analysis, findings were deductively mapped to the RE-AIM framework to support structured interpretation of feasibility outcomes, rather than using RE-AIM as an analytic framework. Three research team members (LH, HH, and MP) read all patient, doctor, and pharmacist transcripts separately to familiarise themselves with the data. One member of the research team (MP) systematically coded each transcript, line by line. A second research team member (LH), reviewed and refined coding. Both MP and LH discussed coding to reach a consensus ensuring interpretative consistency and minimising interviewer bias. Once no new codes could be generated, data sufficiency was achieved as the available data provided adequate and appropriate information to answer the research objectives [32]. Then, categories were developed inductively by grouping similar or related codes to identify patterns in the data [31]. These were gradually refined through repeated review, with subcategories capturing variations within categories. The final categories were then mapped deductively to elements of feasibility from the RE-AIM framework (reach, adoption, implementation, maintenance, and acceptability), integrating the framework at the coding and category development stage, rather than post hoc. Microsoft Excel was used to support quantitative data analysis. Qualitative data were analysed manually, with transcripts coded by hand and codes grouped into categories. We analysed participant cohorts separately to allow for identification of both common and divergent views across stakeholder groups. We did not intend to undertake a formal comparative analysis, but to triangulate findings and provide a broader understanding across all three stakeholder groups.

### Rigour

Rigour was assessed using the pillars of trustworthiness in qualitative research; namely, credibility, transferability, dependability and confirmability [33]. Credibility was ensured during the independent analysis of interview transcripts by different members of the research team, whilst transferability was supported by a detailed description of the study and methods detailed in the protocol. Dependability was addressed through systematic documentation of protocol amendments and analytic decisions. The participant information consent form (PICF) provided for participants to review their transcript on request, to confirm their account of the interview; however, this option was not taken up by any participants. Triangulation was achieved through the inclusion of multiple participant groups (patients, doctors, and pharmacists) and differing interview approaches (structured and semi-structured), reflecting both data source and methodological triangulation. The questions were also tailored for each participant group according to their exposure to the intervention. Investigator triangulation and reflexivity were integrated throughout the study, with pharmacist-researchers (MP, LH, HH) and peers critically reflecting on their potential influence as members of the same health service. Debriefings during regularly scheduled research meetings allowed pharmacists to discuss the interpretation of the findings and reflect on the impressions of the other members of the research team to ground professional biases.

## Results

### Reach and adoption

#### Patients

Whilst a total of 52 patients met the inclusion criteria to participate in the pharmacist-led, multifaceted intervention, only 36 patients were approached for post discharge phone call survey as the remaining 16 patients were discharged back to residential aged care facilities (RACFs). Table 3 presents key quantitative metrics.

**Table 3:** Quantitative metrics

Category	Description	*n* (%)	Comments
Total patients meeting inclusion criteria	Eligible for pharmacist-led, multifaceted intervention	52 (100)	
Approached for post-discharge interview	Excluded 16 patients discharged back to RACFs	36 (69.2)	RACF residents’ medicines managed by facility staff
Consented to participate in follow- up interview	Provided feedback on the intervention	17 (47.2% of those approached)	Patients who did not participate (*n* = 19) declined due to feeling overwhelmed, were discharged early before consent obtained, NESB, limited cognition or no phone access to complete phone follow up interview
Completed follow-up interview	Participated in a phone interview	12 (33.3% of those approached)	Only 12 patients from 17 were contactable by the research pharmacist after discharge, despite multiple phone call attempts to contact patients. A dedicated phone with voicemail facility was used for the purposes of the research.
Provided with the landing page QR code	Access to educational resources	22 (42.3% of total)	15 (68.0%) accessed QR code before discharge
Provided with the question builder QR code	Access to a question-prompting tool	41 (78.8% of total)	17 (39%) accessed before discharge.

Abbreviations: NESB = non-English speaking background,.QR=Quick reference

#### Doctors and pharmacists

All three pharmacists in the selected IPUs participated in the pharmacist-led, multifaceted intervention, and 20 doctors from the general medical and cardiology wards agreed to participate.

### Implementation, maintenance and acceptability

#### Patients

Demographic data on the 12 patients are presented in Table 4, with patient responses to phone surveys conducted one week following their discharge from hospital, presented in Table 5. Of the 12 patients interviewed, 11 (91.7%) agreed that the information provided by the QR code link was helpful, that a GCHHS health care provider discussed medication changes with them, and that they were allowed to ask questions and found the discharge medication records (DMR) helpful. Three-quarters (*n* = 9, 75%) of the patients reported following up with their GP after hospital discharge, with over half of these patients seeing their GP within 1 week.

**Table 4:** Patient demographic data

Variable	Patients, *n* = 12
Age (yrs), mean (SD)	72.3, (5.7)
Male, n (%)	7.0, (58.3)
Number of medications at discharge, mean (SD)	12.0, (4.7)
Predicted risk of medication related harm (PRIME score)	
- Low (score 0–14.9%) - Moderate (score 15.0–29.9%) - High (score > 30%)	5, (42%) 6, (50%) 1, (8%)

**Table 5:** Post-discharge follow-up questions and patients’ responses

Post-discharge questions	Patients’ responses (*n* = 12)	Other comments/feedback
Was the patient information provided helpful whilst in hospital?	• Agree: 11/12 (92%) • Disagree: 1/12 (8%)	The patient who disagreed reported that they did not receive any information.
Did a GCHHS staff member discuss medication changes with you when you were discharged from hospital?	• Yes: 11/12 (92%) • No: 1/12 (8%)	Patient who reported ‘no’, stated that no medication changes were made.
If a staff member discussed medication changes with you, who were they?	• Pharmacist only: 8/12 (67%) • Doctor and pharmacist: 2/12 (17%) • Doctor, pharmacist and nurse: 1/12 (8%) • Couldn’t remember who: 1/12 (8%)	
When medication changes were made in hospital, were you told about them?	• Yes: 7/12 (58%) • No: 4/12 (34%) • N/A: 1/12 (8%)	Patient reporting N/A did not have any medication changes made. Of the 7 patients reporting ‘yes’, 2 patients reported not being told straight away, but just at discharge.
Were you given the opportunity to ask questions?	• Yes: 11/12 (92%) • No: 1/12 (8%)	One patient reported “it was hard to ask questions when you don’t actually know what is happening.”
Did you find the discharge medication record (DMR) listing all your medications, helpful?	• Yes: 11/12 (92%) • No: 1/12 (8%)	Patient who reported ‘no’, stated no medication changes were made.
Did you follow up with your GP after you were discharged from hospital?	• Yes: 9/12 (75%) - Within 1 week: 5/9 (55.6%) - > 1 week: 2/12 (22.2%) - Time frame not specified: 2/12 (22.2%) • No: 3/12 (25%)	

#### Doctors

Of the 20 doctors involved in the pharmacist-led, multifaceted intervention, nine (45%) agreed to participate in structured interviews with a mean interview length of 4.2 min (minimum 3 min, maximum 5 min). Doctors’ demographic data is presented in Table 6.

**Table 6:** Doctor demographic data

Demographic characteristic	*n* = 9
Male, n (%)	4.0, (44.4)
Years since medical registration, mean (SD)	2.2, (1.9)
Current role	
- Intern - Basic physician training (BPT) registrar	6, (66.7) 3, (33.3)
Associated ward	
- General medicine - Cardiology	6, (66.7) 3, (33.3)

Two major categories were identified following analysis of transcripts from doctor interviews:

- Variations in understanding and enactment of the pharmacist-led intervention.
- Perceived value of pharmacist involvement in enhancing discharge processes.

A further six subcategories were identified across the two major categories, presented in Table 7, with the respective feasibility component deductively mapped to these elements (reach, adoption, implementation, maintenance and acceptability) reflected across each of the subcategories.

**Table 7:** Identified categories, subcategories, and exemplar quotes from structured interviews with doctors linked with feasibility components

CATEGORY	SUBCATEGORY	SUPPORTING QUOTES	RE-AIM COMPONENT
1.Variations in understanding and enactment of the pharmacist-led intervention	Variations in documentation of medication plans and rationale for changes	“….we just put them [reasons for medication changes] in the plan.” (Doctor 4) “It could be either…. I think predominantly I did it in the IMR (iEMR) order” (Doctor 6) “most of the time I’d say I would [document] but occasionally not.” (Doctor 9) “Since you presented about this project, it’s been more in the back of my mind to think about using it” [i.e. comments section in Orders] (Doctor 1)	• Implementation
	Factors influencing non-adherence to the intervention.	“it’s unclear why a medication has been ceased or suspended. So, sometimes I don’t know the reason for it” (Doctor 1) “it’s kind of duplicative [recording reason for medication changes in iEMR orders comment section]” (Doctor 5) “……….it’s probably time constraints and time pressure” (Doctor 8)	• Maintenance
2.Perceived value of pharmacist involvement in enhancing discharge processes	Perceived improvements in discharge efficiency with pharmacist involvement	“Hugely……oh, it makes it so much easier…….It definitely helps the flow, it felt like the workflow on those - in those situations, yep” (Doctor 3) “Oh, it definitely aided the workflow.” (Doctor 9) “on the whole, it improves discharge … I think it makes discharges quicker” (Doctor 5)	• Implementation • Acceptability
	Impact of pharmacist-led discharge reconciliation on clinician experience	“[it] would’ve saved me quite a lot of time …………… it facilitated or fostered that sort of teamwork a little bit more. That sort of open communication and as you said, reduces that back-and-forth communication” (Doctor 2) “It definitely reduces some of the cognitive load” (Doctor 8) “I think it improved the workload quite a bit, at least in terms of mental fatigue …I can focus on other things” (Doctor 9) “….we were actually doing nothing and he [hospital pharmacist] was busy so we were like we’ll just do it anyway. So, there are times when we don’t have a lot of workload where it’s probably quicker for us to do it if he’s got a lot to do” (Doctor 4)	• Implementation • Maintenance • Acceptability
	Influence of pharmacist involvement on doctors’ confidence and practice	“….it did fill me with confidence that at least – in most cases during daylight hours, I know that the pharmacist in most cases, if I flagged that a patient is being discharged, that two sets of eyes have been over the discharge reconciliation” (Doctor 2) “….get a good second pair of eyes on the appropriate medications for a patient to be discharged on……….I found it was always easy to follow” (Doctor 7) “I’m still responsible for anything I prescribe, right, so I wouldn’t say that that changes much in terms of confidence” (Doctor 3) “I saw [the pharmacist’s] example of what they would do, and I was like, okay, yeah, sure. That makes sense. I guess it influenced when I have to start a discharge rec myself. Extra points that I can put in there” (Doctor 6)	• Implementation • Acceptability
	Perceived impact on the quality of discharge medication reconciliation	“I think it improves the quality of the discharge…. I think the discharge summary’s better, I think the communication to the GP is better. The communication to the patients better and that there’s less risk of medication errors. ……. I’d say this is the gold standard” (Doctor 5)	• Implementation • Acceptability • Maintenance

#### Category 1 – Variations in understanding and enactment of the intervention

Doctors described variability with how they documented medication plans within the iEMR, with no specific, designated area used consistently. This variation suggests challenges with the consistent implementation of the intervention, reflected in the doctors’ accounts. One doctor reported an increased awareness of the importance of documentation since the intervention started, demonstrating the *acceptability* of the intervention.

Doctors also reported challenges in relation to why they did not routinely document reasons for medication changes, which included being unsure of the reason, time factors, and duplication of documentation elsewhere in the iEMR notes. This lack of a consistent approach presents challenges for *maintenance* or sustainability of the intervention over time.

#### Category 2 – Perceived value of pharmacist involvement in enhancing discharge processes

Doctors described the *acceptability* of the intervention, noting that the pharmacist-led discharge reconciliation component of the intervention made discharge tasks easier and faster. It reduced their workload and cognitive load. Pharmacists’ adherence to discharge reconciliation preparation also demonstrated their delivery of the intervention as intended (*implementation).* Doctors were mindful of supporting pharmacists with preparing discharge medication reconciliations when pharmacists were too busy, which fostered better communication and teamwork (*maintenance)*. Doctors were more confident signing off discharge reconciliations if they knew a pharmacist had prepared this (‘second set of eyes’) and felt comfortable with pharmacists involved in this process, reflecting positive perceptions towards *adoption* and *acceptability*. One doctor felt no change in their level of confidence, given they were ultimately responsible for their prescribing. Another doctor reported that observing a pharmacist preparing a discharge reconciliation provided them with insights into how to follow a structured process, which they planned to emulate in the future (*maintenance).* Participants felt that the quality of the discharge reconciliation was more accurate when prepared by pharmacists than by doctors.

### Pharmacists

Semi-structured interviews were based on three pharmacists taking part in the intervention. Pharmacist interviews were one to two weeks after the intervention had concluded, either face to face or via videoconference, with a mean interview time of 18.7 min (minimum interview time=15 min, maximum interview time=25 min). Demographic data of the pharmacists is presented in Table 8.

**Table 8:** Pharmacist demographic data

Demographic characteristic	*n* = 3
Male, n (%)	3.0, (100.0)
Years since pharmacy registration, mean (SD)	16.3, (9.0)
Current role - Junior pharmacist - Senior pharmacist	0.0, (0.0) 3.0, (100.0)
Associated ward - General medicine - Cardiology	2, (66.7) 1, (33.3)

Four categories were identified:

- Perceptions of doctor documentation.
- Impact of pharmacist discharge medication reconciliation.
- Perceptions of pharmacists’ value-add through discharge medication reconciliation.
- Suggestions to improve the implementation of the model of care.

A further 10 subcategories were identified across the four major categories, presented in Table 9.

**Table 9:** Identified categories, subcategories and exemplar quotes from pharmacist semi-structured interviews linked with feasibility components

CATEGORY	SUBCATEGORY	SUPPORTING QUOTES	RE-AIM COMPONENT
1. Perceptions of doctors’ documentation.	Lack of clarity on documenting reasons for medication changes by doctors.	“Not all the time, [when asked if doctors indicated reasons for medication changes] no………….Sometimes it’s just not clear which we know is an ongoing problem…. it was just a verbal confirmation while they’re on the phone like we normally would.” (Pharmacist 2) “It was a hit and a miss. Some people would say, AKI [acute kidney injury]. Others would just put I think, acutely unwell or something along those lines that is just a blanket that every single prescriber chooses to use without any context or anything in the notes” (Pharmacist 3)	• Implementation • Maintenance
	Ease of locating doctor documentation.	“….documentation was mainly in the ward round notes…to their credit, they still did have documentation, it was just a bit harder to find and it makes our process more difficult sometimes” (Pharmacist 3) “….it could’ve been easier if they had have just documented it on the MAR [medication administration record] when they cease things.” (Pharmacist 2) “Sometimes it’s just not clear which we know is an ongoing problem.” (Pharmacist 1)	
2. Impacts of pharmacist discharge medication reconciliation.	Pharmacist discharge medication reconciliation effects on workflow, workload, efficiency and medication optimisation.	“In terms of the workflow, it absolutely improved, because when you’re waiting for a discharge rec to be done, you can draft your DMR [discharge medication record] up and have an idea of what the plan’s going to be” (Pharmacist 1) “I honestly think when done in the desired way, it was quicker……. I reckon 100 per cent you can optimise better” (Pharmacist 3) “It was not so good in sometimes when you’d gone and done the grind and done it and then you still waited three hours for prescriptions because they were still rounding, and they just didn’t get to it.” (Pharmacist 2) “….it works if the barriers aren’t as much about allied health, I guess. If that MDT [multi-disciplinary team] process isn’t dragging back” (Pharmacist 3)	• Implementation • Acceptability • Maintenance
	Pharmacist discharge medication reconciliation effects on quality and confidence.	“….they’re going to see how it’s done correctly, as opposed to something that’s left half-baked. ………being able to push that off to someone who maybe is a bit more familiar with that sort of process, which will be helpful for them, and then they could focus more on their medicine side of things – not medicines, but the medicine side of things, and other things that they need to learn in their rotation” (Pharmacist 1) (when asked about confidence in taking on the role of discharge reconciliation planning): “Yeah. Yep.” (Pharmacist 2) “Yeah, 100 per cent.” (Pharmacist 3) “I suppose you’re just wondering – you’re hoping that the medical team are looking at your plans before they just sign them off” (Pharmacist 1) “I think my hesitation with that side of things is, I think they read, and I think they acknowledge. I don’t know if they double check the discharge rec for in case I accidentally stopped a home med that I wasn’t meant to” (Pharmacist 3) “somehow you couldn’t have the discharge rec open and still access bloods, notes – whatever it is, that would completely change that workflow going back and forth. That was one of the bigger things I found was frustrating” (Pharmacist 2)	• Maintenance • Acceptability
3. Perceptions of pharmacists’ value-add through discharge medication reconciliation.	Feeling valued, contributing as a team member, working alongside the doctors.	“you could get that extra little bit of rapport with the doctors by saying hey, this is available to do, I can do this for you. …. In terms of that positive interprofessional collaboration point of view, it was good” (Pharmacist 1) “they [the doctors] definitely heard me out. They definitely reviewed the – in the ones that we did, they would review my note, look through my questions and then put it in their note for the day – for the plan” (Pharmacist 3)	• Acceptability
	Mixed feelings of adding value when teamwork and communication broke down.	“Yes [I felt valued] when the teams were committed to the project. No, when the teams weren’t. Because they’d just go and do it willy nilly sometimes anyway. So, valued most of the time, not valued in some other time…. you’ve still got to have that involvement and commitment from the team going forward.” (Pharmacist 2) “….I’d say when done well, there was an impact. When done poorly – not poorly, when done the way it happened where I’d hear about two discharges after the MDT, my workload has increased because they’re now free timewise and have the capacity to do it themselves” (Pharmacist 3)	• Acceptability • Acceptability • Implementation
4. Suggestions to improve the implementation of the model of care.	The need for increased pharmacist presence on doctor teams.	“….having the actual pharmacist as part of the team and part of the rounds would be the most optimal scenario for not only this part of the patient journey, the discharge journey, but also from admission, … That would be the ultimate goal” (Pharmacist 1) “…. in the perfect world, we would have one of us [pharmacist] with every team, and you would be doing it for every patient. You’d be on ward rounds with the team ……. That would just streamline discharge flow so much quicker. …. You’d need so many more of us. Yeah, so that’s the perfect world.” (Pharmacist 2)	• Acceptability • Maintenance
	Consistency is required when doctors document their notes/plans for medication changes.	“I guess it makes it important for the doctors to actually note their thoughts and their plans in the notes a bit clearer. Yeah, we know that sometimes what’s discussed is not always what’s actually written down –…….they’ll know to put a bit more clarity in their notes” (Pharmacist 1) “….it was just a bit harder to find and it makes our process more difficult sometimes” (Pharmacist 2) “it’s important for the doctors to actually note their thoughts and their plans in the notes a bit clearer” (Pharmacist 3)	• Implementation • Maintenance
	Buy in is needed from all doctor and nursing teams.	“Getting the buy-in from them and the importance from them, I think that’d be the biggest thing for me” (Pharmacist 3) “….full commitment from the team, because they won’t fully commit if they’re doing it sometimes and we’re doing it sometimes” (Pharmacist 2)	• Implementation • Maintenance
	The model of care should be better communicated to all staff.	“We should be chatting to the teams and just reiterating, we are doing this tomorrow, this is the plan….they need to be constantly reminded, this is what we’re doing. So, maybe that” (Pharmacist 3) “….more education about it – the biggest limitation from my perspective was that time management of us knowing early…. tell us when you know they are discharging” (Pharmacist 2)	• Implementation • Maintenance

#### Category 1 – Perceptions of doctors’ documentation

Pharmacists expressed a lack of clarity from doctors not routinely documenting reasons for medication changes or locating where reasons for medication changes were documented, demonstrating issues from both an *implementation* and *maintenance* perspective. When doctors documented the reasons for medication changes, pharmacists reported these appeared in the patient’s iEMR ward round note rather than the medication order section.

#### Category 2 – Impacts of pharmacist discharge medication reconciliation

Pharmacists noted the *acceptability* of the intervention with positive feedback around streamlining medication reconciliation, supporting faster discharge planning, considering overall workflow, workload, efficiency and medication optimisation (*implementation)*. One pharmacist expressed mixed opinions in terms of the impact on their workflow, acknowledging that pharmacist-led discharge reconciliation streamlined overall workflow, but could remain slow if doctors did not action the pharmacist’s work if the medical team was still on their ward round (*maintenance)*. Pharmacists reported computer obstacles with the iEMR system itself, presenting challenges to workflow and sustainability (*maintenance)*. Another pharmacist noted that despite their best intentions in organising discharge medication reconciliation for a patient declared medically fit for discharge, the process could still be delayed if allied health teams deemed the patient unfit for discharge (*maintenance)*.

Pharmacists noted doctors had the opportunity to observe how a discharge medication reconciliation should be prepared correctly, presenting a learning opportunity for doctors, which supported the sustainability of the intervention (*maintenance)*. Since pharmacists saved doctors’ time, doctors could also focus on learning associated with their medical rotation (*acceptability)*.

Pharmacists reported feeling confident in undertaking discharge medication reconciliation after receiving initial training, which demonstrated both *acceptability* and *maintenance* domains. However, pharmacists expressed concerns around fidelity, querying whether doctors checked the medication discharge reconciliation they had prepared, as opposed to just signing them off (*implementation)*. There was a sense of concern about not having a second check to prevent medication errors.

#### Category 3 – Perceptions of pharmacists’ value-add through discharge medication reconciliation

Intervention *acceptability* was articulated by pharmacists, perceiving they added value to the discharge process, developing rapport and being heard by the doctors, and working together as a multiprofessional team. One pharmacist noted challenges with *implementation* and *acceptability* domains when working alongside doctors who had not fully committed to the intervention.

#### Category 4 – Suggestions to improve the implementation of the model of care

Pharmacists reported that increased pharmacist presence on medical teams would better integrate the model of care into daily practice, reflecting the sustainability of the intervention *(acceptability* and *maintenance)*. Addressing fidelity issues by requiring doctors to document reasons for medication changes in a consistent manner in the medical records would further improve clarity *(implementation)*. Ensuring ‘buy in’ from all health care professionals within the hospital would ensure greater model consistency and long term sustainability, with clear communication to all when preparing a patient for discharge. *(implementation* and *maintenance*). Table 10 presents an overarching summary, mapping the four components of the pharmacist-led, multifaceted intervention to the RE-AIM framework.

**Table 10:** Multifaceted intervention aligned to RE-AIM domains

Component	Reach	Adoption	Implementation	Maintenance	Acceptability
Patient engagement	47% consented 68% used QR code	Patients engaged with tools	QR codes were integrated into discharge workflow	Sustainability depends on patient IT literacy/support	92% felt included in medication decisions
Doctor documentation	20 doctors involved	Variable uptake across teams	Inconsistent rationale recording medication changes at times	Training and clear protocols required	Mostly positive: pharmacist-led reconciliation helpful, minor concerns re: duplication
Risk stratification	52 patients eligible, High risk patients identified	Fully adopted by pharmacists	Validated risk prediction tool applied	Dependent on pharmacist resourcing/capacity	Viewed as targeted and efficient
Discharge reconciliation	3 pharmacists, 52 patients eligible	Adopted by all wards involved	Improved workflow, reduced duplication	Requires funding, staffing, IT support	Strongly positive: valued by staff and patients, workload/communication challenges noted

Feasibility outcomes:

Intervention viewed favourably

Positive staff and patient perceptions

Improved workflow and inclusion

Barriers: documentation, workload, IT

Supports refinement and scale-up

## Discussion

### Summary of findings

This study evaluated the feasibility of a pharmacist-led, multifaceted intervention to improve medication information transfer at discharge from hospital. Overall, the intervention was acceptable to patients who reported positive discharge experiences, finding information helpful and having opportunities to ask questions. Nine doctors and three pharmacists described that the pharmacist-led discharge medication reconciliation process streamlined workflows and reduced doctors’ cognitive burden, aligning with previous literature [34–36]. Findings were mapped to the RE-AIM framework at the discussion stage to organise and structure feasibility outcomes, rather than as an analytic framework guiding coding. Overall, the intervention appeared feasible based on assessment against the RE-AIM framework, although further work is needed to refine *implementation* and *maintenance* domains to increase long-term intervention success.

### Intervention benefits: workflow, collaboration and patient experience

#### Workflow and workload

Doctors and pharmacists described that pharmacist-led discharge medication reconciliation reduced workload and improved workflow, consistent with previous studies of pharmacist-led reconciliation models [35]. Time-based analysis from this study [22] demonstrated a reduction in discharge preparation and clinical review (7.44 min for intervention patients vs. 17.10 min for control patients), suggesting improved efficiency, aligning with other literature using collaborative pharmacist medication prescribing [35]. Whilst our findings demonstrate workflow benefits, timely discharge is influenced by multiple factors beyond medication reconciliation, including input from other members of the health care team [36]. This highlights that, despite pharmacist collaboration streamlining workflow processes, broader system-level factors continue to impact patient discharge processes.

#### Collaboration

The pharmacist-led discharge medication reconciliation was associated with enhanced collaboration. Doctors were able to see pharmacists undertake accurate discharge medication reconciliations and increase their own knowledge base. Doctors reported increased confidence in discharge medication reconciliation, supported by pharmacists, whilst pharmacists expressed enjoyment at working to their full scope, adding value to the discharge process. These findings align with previous research demonstrating that role clarity, trust and shared responsibility have been positively associated with effective physician/pharmacist collaboration [37] and an enhanced sense of team [38]. The intervention also provided a positive learning environment, with doctors reporting greater insights into how to improve preparing a discharge medication reconciliation, through their interactions with the pharmacist. Other pharmacist-led medication management interventions involving pharmacists supporting doctors in managing their workload have shown positive impacts in health care team satisfaction, aligning with the sentiments in the current study [39].

#### Patient experience

Patients reported positive experiences and generally felt included in medication related matters, receiving information about medicine changes and having an opportunity to ask questions. This suggests that the intervention facilitated increased patient engagement and communication prior to discharge. Our intervention fostered an environment to enhance patient knowledge, shared understanding and therapeutic alliances, all of which has been show to improve health and well-being [40, 41] and reduce hospitalisations and health status [42].

### Implementation challenges in real-world practice

There was a lack of consistency with one of the intervention components requiring doctors to document reasons for medication changes in the correct section of the ieMR. Consequently, pharmacists struggled to locate the medical teams’ intentions around medication management decisions. These findings suggest that the variability in doctor documentation practices reduced the consistent implementation of the intervention. Previous research has shown that effective use of electronic health records requires practitioners to have a clear understanding of their responsibilities, including documentation [43]. Our study highlighted that inconsistencies in doctors’ documentation resulted in challenges embedding the intervention into day to day practice. Poor adherence to an intervention undermines fidelity and risks rejecting an intervention that may have been successful if implemented properly, so addressing this variability is crucial for success [44].

While the intervention effectively fostered a sense of inclusion among patients, their limited use of the QR codes—intended to prompt engagement around medication-related questions is unsurprising. This likely reflects known barriers associated with patients navigating information technology (IT) to improve health care literacy and suggests that patients need support when using the application. Extended patient training and support in using the QR technology may have yielded greater results, especially considering that the patients included in the intervention were older patients [45, 46].

### Sustainability and scalability considerations

Sustaining and scaling the intervention will require system level support, with a focus on funding, workforce capacity and workflow integration. Whilst pharmacist-led discharge medication reconciliation led to improved workflow and reduced doctor workload, there is a risk that the additional work is absorbed by pharmacists, highlighting the need for workforce planning. Ideally, outcomes demonstrating the efficacy of the intervention would assist in developing a business case for establishing recurrent funding to support and sustain the model long term. Our previous publication reporting on the efficacy of the intervention demonstrated a reduction in length of stay [22]. Cost savings from reduced length of stay could be used to generate additional funding to scale up and sustain the intervention long term [47].

Addressing implementation challenges (e.g., variability in doctor documentation) has been shown to increase the success of implementing a more sustainable intervention [48]. Developing a structured, interprofessional, dedicated training program for the pharmacist-led discharge medication reconciliation process, with clear responsibilities assigned and role delineation, could be one approach towards managing implementation issues.

Digital tool refinement to enhance pharmacist workflow whilst preparing discharge reconciliation would also improve efficiencies. Regular review or auditing of the intervention could be undertaken on a 6-monthly or annual basis, with the development of key performance indicators to monitor and improve the intervention.

Improving equity of access for patients with cognitive impairment, or from culturally and linguistically diverse backgrounds, should also be considered to optimise patient engagement. This may require modification of QR-code tools into languages to suit people from non-English speaking backgrounds, non-digital education options for patients with barriers to information technology, or information for carers or family members.

### Implications for practice and future research

This study demonstrates the feasibility of collaborative pharmacist medication prescribing within the context of patient discharge on medical wards and potentially lends itself to further roll out across surgical and other subspecialities. However, successful implementation will require standardisation of documentation practices, improved digital integration and interprofessional training to embed the intervention into daily practice. Future research should consider the clinical and cost-effectiveness in larger, multi-site studies, including time to discharge summary completion, medication related harm rates within 30 days of discharge and cost effectiveness analyses. Our next step is to discuss our outcomes with GCHHS management to determine which of the pharmacist-led, multifaceted components to incorporate into a larger trial.

### Strengths and limitations

Strengths of the study included the use of a mixed-method design incorporating patient, pharmacist and doctor perspectives, allowing triangulation of findings across multiple stakeholders. Application of the RE-AIM framework provided a structured approach to evaluate each of the four components of the multifaceted intervention across key feasibility domains. Conducting the study within busy inpatient wards as part of routine clinical practice, also allowed us to test the practicality of the intervention, and the ease with which it would be implemented in a real-world, clinical setting. Limitations include a small sample size of patients and health care professionals, with the intervention only trialled on three wards, limiting the external validity to other health care settings.

We acknowledge that the small sample size of participants who agreed to brief or semi-structured interviews may have also influenced internal validity. However, selection bias was mitigated, given that all invited clinicians proceeded to participate in brief or semi-structured interviews. We also acknowledge that the mean interview length with doctors, of 4.2 min, is unlikely to provide an in-depth understanding. However, we were cognisant of doctor time challenges, given we were interviewing them during working hours, with a focus on capturing perspectives of specific aspects of the intervention, rather than generating rich, in-depth qualitative data. Social desirability bias was also managed through the use of open-ended questions during surveys and interviews. Another limitation of this feasibility study was the challenge of estimating the eligible population of doctors from which our sample was drawn, given there was transient movement of doctors across multiple wards, with rotating junior medical staff, varying roster allocations and cross-cover arrangements. For this reason, we adopted a pragmatic recruitment approach based on clinician availability and ward presence. We acknowledge the limitations this imposes when considering the reach of the intervention. For future, larger scale studies, we would plan to more accurately monitor the number of eligible doctors exposed to the intervention, so we could more easily quantify the reach and adoption of the doctors.

Despite the limitations associated with the use of data collection method (brief and semi-structured interviews), reflexivity and triangulation across patient, doctor and pharmacist responses minimised potential bias from pharmacist researchers’ interpretation of results. Findings were mapped to the RE-AIM framework at the discussion stage to organise and structure feasibility outcomes, rather than as an analytic framework guiding coding. We acknowledge that this mapping reflects the authors’ interpretation of alignment with RE-AIM domains. Future studies should also consider using a structured fidelity assessment assessing adherence, exposure, reach, quality of delivery and responsiveness more formally, to provide a comprehensive evaluation.

## Conclusion

The pharmacist-led, mutlifaceted intervention was acceptable to patients, doctors, and pharmacists and its feasibility in improving medication information transfer at hospital discharge is promising. Patient feedback highlighted consistent communication from health care professionals discussing medication changes, with opportunities for patients to ask questions. The intervention also facilitated timely patient follow up with GPs following discharge from hospital. Pharmacist-led medication reconciliation improved workflow and reduced workload, whilst providing mutual learning opportunities for both doctors and pharmacists. These findings generate useful hypotheses for refining and testing the digital components of the intervention to enhance accessibility and engagement. However, implementation challenges relating to workflow integration and documentation, demonstrate the need for system-level support. A future implementation trial powered to test clinical effectiveness and real-world uptake of the pharmacist-led intervention with cost-effectiveness analysis would be the next logical step.

## Supplementary Information

Below is the link to the electronic supplementary material.


Supplementary Material 1


## Data Availability

De-identified datasets used and/or analysed during the current study are available from the corresponding author on reasonable request.

## Declarations

### Generative AI and AI-assisted technologies in the manuscript preparation process

During the preparation of this work the author used ChatGPT in order to assist with the design and layout of Tables 3 and 10, and minor revisions and editing. After using this tool/service, the author(s) reviewed and edited the content as needed and take(s) full responsibility for the content of the published article.
