# Supplementary material for: Implementation of a pharmacist‑led discharge intervention to improve medication information handover at hospital discharge: a non-randomised feasibility study
Source: BMC Health Serv Res. 2026 Jun 17;26:1199. doi: 10.1186/s12913-026-14940-1 (PMC13527957; doi:10.1186/s12913-026-14940-1)
Supplement: Supplementary file 1 [file 12913_2026_14940_MOESM1_ESM.pdf]

Interview Guide

**Consumer**
Was the patient information provided helpful while in hospital?<br>Did a Gold Coast Health staff member discuss medication changes with you?<br>• If so, who was it? Nurse, Pharmacist, Doctor<br>When changes to your medication were made in hospital, were you told about them?<br>• Were you given the opportunity to ask questions?<br>Did you find the Discharge Medication Record helpful?<br>Did you follow up with your General Practitioner after discharge?<br>• If so, how many weeks after discharge?<br>Did you feel more confident about your medications compared to previous discharges?<br>• Yes, No or N/A<br>Did you use the patient information QR code at home?
**Gold Coast Health Staff**
**Pharmacist**
Was the PRIME score helpful?<br>• What did you do with this score?<br>• How did it impact medication handover for the patient?<br>• Did the doctor's indicate reasons for changed, ceased or new medications?<br>• Did they use the iEMR 'cancel reason' and add a comment?<br>o Was it easy to use this function?<br>• Did they document in clinical notes?<br>• Did they verbally tell you?<br>Discharge reconciliation planning:<br>• What was the impact on workflow?<br>• What was the impact on workload?<br>• Did you feel confident taking on this role?<br>• Did you feel confident in planning the discharge reconciliation?<br>• Did you feel valued by the team when discussing the discharge reconciliation?<br>• Did this help facilitate the timeliness of discharges?<br>Did patient's ask about the patient information QR code?
**Doctors**
Did you document medication changes?<br>• How did you document these changes?<br>Discharge reconciliation planning:<br>• What was the impact on workflow?<br>• What was the impact on workload?<br>• Did you feel more confident about doing discharge reconciliations?<br>• Did you feel comfortable with the pharmacist's involvement in the discharge process?<br>• Did this help facilitate the timeliness of discharges?
